# Spontaneous Transvaginal Intestinal Evisceration Two Years after Vaginal Hysterectomy, a Case Report

**DOI:** 10.3390/medicina60091388

**Published:** 2024-08-24

**Authors:** Andrej Omejc, Vid Janša, Borut Kobal, Matija Barbič

**Affiliations:** 1 Faculty of Medicine, University of Ljubljana, Vrazov trg 2, 1000 Ljubljana, Slovenia; andrej.omejc1@gmail.com (A.O.); vid.jansa@gmail.com (V.J.); borut.kobal@kclj.si (B.K.); 2 Department of Obstetrics and Gynecology, University Medical Centre Ljubljana, Šlajmerjeva 3, 1000 Ljubljana, Slovenia

**Keywords:** transvaginal evisceration, small bowel evisceration, vaginal surgery, case report

## Abstract

Vaginal evisceration is an exceedingly rare and poorly documented complication following vaginal hysterectomy. Prompt detection and surgical intervention are critical to prevent severe complications such as bowel ischemia, perforation, and secondary sepsis. We present the case of an 84-year-old woman with a history of vaginal hysterectomy two years prior, who presented with acute abdominal pain and a significant portion of her small bowel protruding through a defect in the vaginal vault. The patient was urgently transferred to the operating room, where the loops of the small bowel were manually reduced through the vaginal defect. As the bowel appeared viable, no resection was required. The etiology of this condition is unclear and likely multifactorial. Various surgical approaches, including laparoscopic, abdominal, transvaginal, and combined techniques, have been described, all offering comparable outcomes. Therefore, the choice of surgical procedure should be tailored to the patient’s clinical presentation.

## 1. Introduction

Vaginal evisceration refers to the extrusion of abdominal viscera through a defect or rupture in the vaginal wall [1]. It is an exceptionally rare surgical emergency that necessitates immediate intervention to prevent severe complications, including bowel necrosis, perforation, and secondary sepsis. Only a limited number of cases have been documented in the literature [2]. Typically, the disease presents subtly, with imaging showing small bowel obstruction or diagnostic laparotomy revealing a small bowel loop herniating through the vagina [3]. However, in rare instances, it presents more dramatically, with exposed bowel loops protruding through the vagina, causing significant abdominal discomfort and posing an acute risk of bowel ischemia [1,3].

Vaginal evisceration most commonly occurs in post-menopausal women with a history of vaginal surgery, particularly those with concomitant pelvic organ prolapse, which are the two primary risk factors for this condition [2]. In most reported cases, the onset of vaginal evisceration is associated with a specific trigger event that leads to a sudden increase in intra-abdominal pressure, such as coughing, constipation, or recent trauma or surgery, which can provoke vaginal rupture and subsequent intestinal evisceration in patients with predisposing risk factors that weaken the vaginal wall [2].

In this case report, we present a post-menopausal woman with a history of vaginal hysterectomy two years prior, who was admitted to the hospital with severe abdominal pain and exposed loops of the terminal ileum protruding through the vagina. Notably, the patient reported no trigger events that could have precipitated the onset of this condition.

## 2. Case Presentation

An 84-year-old patient presented with a sudden onset of abdominal pain and a sensation of something protruding from her vagina. The pain was described as severe, constant, and localized to the hypogastric region. The patient reported passing urine and stool that morning without additional straining. She did not recall any coughing episode or event that could have increased intra-abdominal pressure. Her surgical history revealed a vaginal hysterectomy (Crossen technique) performed two years earlier due to uterovaginal prolapse. Six months after the surgery, she experienced a recurrence of vaginal vault prolapse (enterocele), which persisted until the present day. She was initially treated with pessary placement, but due to poor tolerance, the pessary was removed. The patient had a history of two uncomplicated term spontaneous vaginal deliveries, with menopause at 50 years of age. Additionally, the patient was undergoing treatment for hypothyroidism following a thyroidectomy.

**Figure 1:**
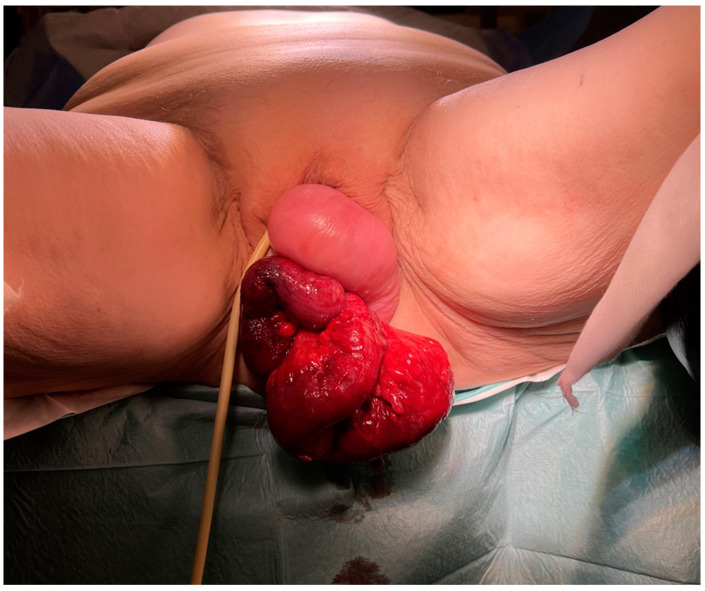
Edematous and hyperemic small bowel transvaginal evisceration due to spontaneous perforation of vaginal vault 2 years after vaginal hysterectomy.

Upon admission, the patient was in significant pain but remained hemodynamically stable with normal vital signs; she was afebrile and pale. Her abdomen was diffusely tender. A perineal examination revealed prolapse of the vaginal vault with a 3 cm perforation of the vaginal wall, through which a significant portion of the terminal ileum was protruding (Figure 1). The bowel loops appeared dusky and hyperemic, though there were no obvious signs of necrosis. The patient was admitted to our department and prepared for emergency surgery to preserve intestinal viability and avoid resection if possible.

The surgical procedure began with a lower midline incision. Upon inspection, the abdominal cavity appeared normal. The protruding bowel loops were manually reduced back into the abdominal cavity through the vaginal wall defect. A consultant abdominal surgeon inspected the bowel loops once more, confirming their hyperemic state but noting no obvious signs of necrosis, which ruled out the need for bowel resection. After inspection, the bowel was covered with wet sterile gauze. A large portion of the prolapsed vaginal tissue was resected. The rectum’s consistency was assessed through manual inspection and dye injection. The remaining vagina was closed, and the vaginal vault was fixed to the ligamentum rotundum, with obliteration of the Douglas pouch. Additionally, a bilateral adnexectomy was performed. Two drains were placed in the abdominal cavity, and resected tissue samples were collected for pathological examination. Perioperative metronidazole and gentamycin antibiotic combination was administered.

At the regular follow-up examination six months after the surgery, the patient did not report any issues. No abnormalities were observed during the gynecological examination.

## 3. Discussion

Transvaginal intestinal evisceration is a rare surgical emergency, with only a limited number of cases documented in the literature. Approximately 70% of these cases occur in postmenopausal women [2], likely due to vaginal wall atrophy associated with hypoestrogenism, chronic tissue devascularization, and pelvic floor weakness. This triad results in a vascularly compromised, thin, scarred, and shortened vagina that is more prone to rupture [4].

The primary risk factors in this age group include previous gynecological surgery and pelvic organ prolapse. The incidence of vaginal evisceration following a pelvic operation is reported to be 0.032% [5], with vaginal hysterectomy being the leading cause [4]. Vaginal evisceration after vaginal hysterectomy typically results from weakness at the vaginal apex, often due to postoperative complications (such as wound infections or hematomas), premature resumption of sexual activity, advanced age, and improper surgical techniques during the initial operation. Other contributing factors include delayed wound healing due to radiation therapy, chemotherapy, chronic steroid therapy, or electrolyte imbalances (such as hyponatremia); inadequate vitamin levels due to malnutrition; untreated chronic conditions (like chronic renal failure and diabetes); and pulmonary diseases that impair tissue oxygenation.

The median time from vaginal hysterectomy to evisceration is reported to be 20 months, with the vaginal vault being the most common site of evisceration [4]. Another significant risk factor is pelvic organ prolapse, particularly when associated with enterocele, as the prolapse further stretches the atrophic vagina, increasing the risk of rupture [4]. Risk factors for postoperative vaginal vault prolapse are preoperative prolapse and sexual activity.

In the premenopausal population, vaginal evisceration is even rarer and is most often associated with instrumentation, obstetric injury, or coital trauma, which can cause lacerations of the vaginal wall [2].

The terminal ileum is the most common organ to protrude through the vaginal defect, though cases involving the omentum, colon, fallopian tubes, and the appendix have also been reported [6].

The onset of evisceration in patients with risk factors is often triggered by a sudden increase in intra-abdominal pressure, such as during a coughing episode, constipation, ascites, or recent abdominal surgery [2].

We present a case of a postmenopausal patient with additional risk factors, including chronic vaginal vault prolapse and a history of vaginal hysterectomy. In this patient, evisceration occurred 21 months after vaginal hysterectomy, with the vaginal vault as the site of protrusion and the terminal ileum as the protruding organ. Notably, the patient reported no specific triggers that could have caused the vaginal wall to rupture.

Symptoms of vaginal evisceration and their severity depend on the size of the defect and the nature of the protruding tissue. Common symptoms include the observation of protruding tissue from the vagina, accompanied by vaginal pain, bleeding, and discharge. These symptoms typically occur following triggers such as sexual activity (especially when recent vaginal surgery has been performed), vaginal instrumentation, or situations that elevate intra-abdominal pressure [4]. In most cases, the symptoms are acute but subtle, without the clinical findings typical of an acute abdomen. When this is the case, diagnosis may be aided by imaging showing small bowel obstruction or diagnostic laparotomy revealing bowel protrusion through the vaginal defect. Rarely, as in our case, it manifests dramatically with large loops of small bowel prolapsing through the vagina.

Transvaginal small bowel evisceration is associated with a 6–8% mortality rate and a 15–20% morbidity rate [2], primarily due to complications such as intestinal ischemia, gangrene, and abdominal sepsis caused by damage to the vascular integrity of the small bowel from mesenteric laceration, which often accompanies transvaginal bowel evisceration [4,7,8].

The early detection and surgical management of vaginal evisceration are crucial to prevent small bowel ischemia, which may necessitate resection, and to avert the development of sepsis and systemic inflammatory response syndrome due to bowel necrosis [3]. Initial management should focus on stabilizing the patient, managing fluid status, administering broad-spectrum antibiotics, covering the protruding viscera with sterile gauze, and promptly transferring the patient to the operating room for definitive repair [1].

The primary goal of surgery should be a thorough examination of the protruding bowel loops, assessment of their viability, and, when necessary, resection of any non-viable bowel. Given the rarity of this condition, there is no unified consensus on the optimal surgical technique, and both vaginal and abdominal approaches have been utilized. If the bowel is easily reducible, has not suffered prior radiation injury, and there is no evidence of an acute abdomen, a vaginal approach may be considered [3]. However, its limitations include the surgeon’s inability to thoroughly inspect the bowel and mesentery. Additionally, if bowel repair is required, the vaginal approach is not appropriate.

A combined laparoscopic and vaginal procedure has also been reported as beneficial, as it allows for proper inspection of the abdominopelvic viscera before repairing the vaginal defect [9,10]. However, in most cases, including ours, intraperitoneal repositioning of the eviscerated intestine is not possible due to the condition of the affected loops and the location of the vaginal defect [3]. In such cases, an abdominal approach is necessary. This procedure involves making a midline vertical incision, followed by a comprehensive inspection of the entire intestine and mesentery, from the Treitz ligament to the cecum, with an assessment of any lacerations and vascular injuries. Suspicious areas of bowel should be resected, followed by lavage of the peritoneal cavity. The repair of the vaginal defect should involve removing all necrotic tissue from the vaginal cuff and supporting structures, leaving only fresh, viable tissue [2,3,11,12].

Since both vaginal and abdominal approaches have comparable rates of recurrence and complications, the choice of approach should be individualized based on the patient’s condition and managed by a multidisciplinary team [2,3,4].

Given the extreme rarity of this condition, establishing definitive principles for the prevention of vaginal evisceration is challenging. Since vaginal surgery is the most significant risk factor, it is advisable to avoid repeated surgical procedures that alter vaginal anatomy and tissue integrity. Additionally, pelvic support defects should be addressed, and hypoestrogenism should be managed effectively [4].

To prevent vaginal evisceration, several surgical considerations should be taken into account when performing a vaginal hysterectomy. If an accompanying enterocele or cystocele is present, it should be repaired during the initial hysterectomy. The surgeon must carefully weigh the advantages and disadvantages of leaving the vaginal vault open. Furthermore, the importance of properly closing the vaginal vault using appropriate techniques and suture materials cannot be overstated. The technique used for anastomosing the stumps of the supporting pelvic ligaments to the angles of the vagina should also be emphasized [4].

## 4. Conclusions

Vaginal evisceration is an exceptionally rare complication following vaginal hysterectomy. Early detection and prompt surgical management are crucial to prevent bowel ischemia, necrosis, and the resulting risks of sepsis and death. It most commonly occurs in postmenopausal women, with a history of vaginal surgery and concurrent pelvic organ prolapse being the two leading risk factors. Although vaginal evisceration typically occurs shortly after the initial vaginal surgery, it can also manifest much later, as in the case of our patient, where it occurred two years after the vaginal hysterectomy. The choice of the optimal surgical approach should be tailored to the individual patient, based on their presentation and overall condition.

## Data Availability

The data presented in this study are available on request from the corresponding author.
